# A study on changes of the resting-state brain function network in patients with amnestic mild cognitive impairment

**DOI:** 10.1590/1414-431X20198244

**Published:** 2019-04-29

**Authors:** Jun Min, Xu-Xin Zhou, Feng Zhou, Yu Tan, Wei-Dong Wang

**Affiliations:** 1 Department of Rehabilitation, Third Affiliated Hospital of Nanchang University, Jiangxi, China; 2 Department of Biomedical Engineering, School of Measuring and Optical Engineering, Nanchang Hangkong University, Nanchang, China; 3 Department of Neurology, Third Affiliated Hospital of Nanchang University, Nanchang, China; 4 Department of Neurology, Shenzhen Hospital of Southern Medical University, Shenzhen, China

**Keywords:** Amnestic, Mild cognitive impairment, Function network, Resting state, Functional magnetic resonance imaging

## Abstract

This study aimed to explore the structural and functional characteristics of the neural network of resting-state brain activities in patients with amnestic mild cognitive impairment (aMCI) by functional magnetic resonance imaging (fMRI) technology. Resting state fMRI scanning was performed on 10 clinically diagnosed aMCI patients and 10 healthy volunteers, and the difference in the resting-state brain activities between aMCI patients and healthy volunteers was compared using the brain function network regional homogeneity (ReHo) analysis method. Results of the ReHo analysis of aMCI patients and healthy volunteers revealed that the ReHo value significantly increased in the posterior cingulate gyrus region, medial frontal lobe, medial cortex of the prefrontal lobe, and part of the parietal lobe. Compared with the normal elderlies, ReHo decreased in aMCI patients in the left temporal lobe (middle temporal gyrus and inferior temporal gyrus), left parahippocampal gyrus, occipital lobe, lingual gyrus, precuneus, and other regions while ReHo increased in regions of the right frontal lobe (inferior frontal gyrus), left superior temporal gyrus, precentral gyrus (frontal lobe), right thalamus, left fusiform gyrus, and other regions. In the resting state, there may be regional abnormalities in brain functional areas in aMCI patients, which may be associated with cognitive impairment.

## Introduction

Alzheimer's disease (AD) is the most common cause of senile dementia. It is characterized by progressive memory loss, which gradually impacts a patient's cognition and non-cognitive functions. It has been reported that approximately 10–15% of patients with mild cognitive impairment (MCI) develops into AD per year (1). The clinical manifestations and prognosis of MCI patients are heterogeneous. MCI patients not only exhibit memory impairment, but also exhibit mild cognitive impairments of attention, executive ability, and other cognitive functions. Therefore, Petersen et al. (2) proposed the concept of amnestic MCI (aMCI) based on clinical evaluations. Episodic memory disorder is the outstanding manifestation of aMCI, and the annual conversion rate of aMCI to AD is 10 times that of the incidence of AD in the normal population. Therefore, aMCI is considered the precursor phase of AD; if early diagnosis and treatment are performed at this stage, patients' quality of life will be improved.

Brain functional magnetic resonance imaging (fMRI) is mainly performed based on the principle of blood oxygenation level dependent (BOLD) contrast enhancement. Before MCI progresses into AD, BOLD-fMRI is able to detect changes in brain functions, and it is an important technique for studying the neural mechanism of MCI (3). For example, Qi, et al. (4) found that diabetes mellitus type 2 (T2DM) patients with aMCI showed abnormal functional connectivity patterns and decreased white matter integrity, making fMRI a potential diagnostic tool for early detection of AD in elderly T2DM patients. Another study used fMRI to evaluate the resting-state brain function. The authors demonstrated that changes in functional brain networks involved in cognition, such as episodic memory and visual cognition in aMCI, were altered, and provided new insight into understanding the important subtype of aMCI (5).

In the present study, resting fMRI technology was used to compare the ReHo between normal elderlies and aMCI patients, aiming to explore the homogeneity and differences of brain functional activities in the resting state in both populations, and to provide a candidate region of interest for subsequent study on brain functional connectivity, as well as a basis for the early diagnosis of aMCI.

## Material and Methods

### Research subjects

#### aMCI group

Ten patients who were diagnosed with aMCI by neuropsychological testing and imaging examination at the Third Affiliated Hospital of Nanchang University from September 2010 to December 2011 were selected. According to the diagnostic criteria of aMCI proposed by Petersen et al. in 2001 (1), inclusion criteria were as follows: 1) patients between 65–75 years old with Han nationality and right-handed; 2) patients whose chief complaint was memory loss, and this symptom had been lasting for more than six months; 3) patients with normal general cognitive function and Mini Mental State Examination (MMSE) scores between 24–28 points; 4) patients whose ability of daily life was preserved and had ability of daily living (ADL) scores of <26 points; 5) cognitive disorders did not meet the diagnosis of dementia, and the clinical dementia rating (CDR) scale score was 0.5 points. Exclusion criteria were as follows: 1) patients with a Hachinski ischemic score of >4 points and a history of acute cerebral vascular disease in the past three months; 2) patients with active epilepsy; 3) patients with Hamilton Depression Scale (Hamilton) scores of >12 points and had a history of mental illness; 4) patients with the presence of liver and kidney dysfunction that affected cognitive function, deficiencies of folic acid and vitamin B12, abuse of drugs and alcohol, and severe infection; 5) patients with advanced, serious, or other unstable diseases, which affect the assessment of brain function or cognitive function. A total of 10 patients were enrolled including five males and five females, with an average age of 69.80± 2.658 years. The average years of education was 13.20± 1.135, and the MMSE score was 25.90±0.738 points.

#### Normal control group

Ten normal elderlies with matched age, gender, and education level were enrolled in this study and underwent physical examinations at the same period. The inclusion criteria were: 1) subjects between 65 to 75 years old with Han nationality and right-handed, who had good overall health conditions and had no nervous system disease; 2) no memory complaints and clinical manifestations; 3) normal overall cognitive function and MMSE scores of >27 points; 4) ADL scale scores of <26 points; 5) no cognitive impairment and a CDR scale score of zero point. Five males and five females were enrolled with an average age of 69.90±2.601 years, 13.60±1.713 average educational years, and an average MMSE score of 29.30±0.823 points.

There was no significant difference in age, educational years, or gender between the aMCI group and the control group (P>0.05). However, there was a significant difference in MMSE scores between these two groups (P<0.001). This research was approved by the Medical Ethics Committee of Nanchang University. All subjects and their families were informed of the research content, were willing to cooperate with the trial, and provided written informed consent.

### Research methods

#### MR scanning

A GE-3.0T superconducting magnetic resonance scanner (USA) was used. During the whole process, subjects were instructed to remain in the resting state, keep awake, take the supine position, close their eyes, try to relax, and refrain from moving. The head was fixed, and the active and passive relationship between the head and other parts were maximally reduced. At the same time, any brain activity for thinking by the subjects was avoided. The resting state scan parameters were: gradient echo planar imaging sequence, repetition time (TR)=2,000 ms, echo delay time (TE)=30 ms, flip angle=90°, field of view (FOV)=25.6, array=64×64, slice thickness=3 mm, slice gap=1.0 mm, number of slices=32, and total time=6 min and 40 s.

#### Data processing

The statistical parametric mapping software running on the 2010 Matlab (MathWorks, USA) was used. The format of the acquired imaging data was transformed using the MRIcro software package (http://www.mricro.com), alignments at the dimensions of space and time and the correction of head movements were performed using SPM8 and DPARSF software (Institute of Psychology, Chinese Academy of Sciences, China), and head motion parameters during the scanning were obtained at the same time. The first 10 images were excluded, considering that a certain amount of time was needed to make the magnetic field steady, as well as to allow the subjects to adapt to the environment. The remaining 190 images were included in the analysis processes. These images met the criteria, and were normalized using the EPI template, and re-sampled to 3×3×3 mm^3^. Spatial smoothing was performed using a 4-mm Gauss kernel with a full width at half maximum to reduce the spatial noise and anatomical differences in the structures of these subjects. Then, the linear drift was removed by removing the six head dynamic parameters using the linear regression method. Next, wave filtering was performed (0.01<f<0.08 Hz).

ReHo was analyzed after low frequency wave signals were obtained. The ReHo analytical method is a data analysis based on the calculation of Kendall's coefficient of concordance (6 [Bibr B07]). It is used as an index to measure the homogeneity of time-series changes between adjacent voxels, to analyze the homogeneity of spontaneous brain activity using whole brain functional imaging data. The homogenous spontaneous activity in the brain is secondary to nerve activity. This method can be used to measure the homogeneity of local BOLD signals, which reflects the homogeneity of nerve activity in the brain.

The two-sample *t*-test was used for inter-group analysis to detect the difference in ReHo values in brain regions between the aMCI group and control group (P<0.05 indicated a statistically significant difference). The size of the mass was more than 20 voxels (uncorrected). A statistical parametric map was added onto the glass map, and the regions with significant differences in these two groups were described using the known neuroanatomical markers.

## Results

**Figure 1.**
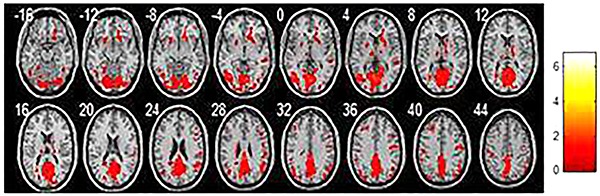
Average regional homogeneity in the normal elderlies. Each image represents a slice, with 4 mm distance from the other. 0 is the 0 position of space coordinates. The color bar indicates energy intensity.

**Figure 2.**
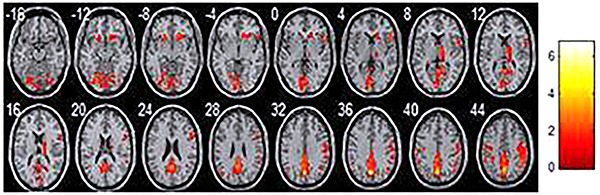
Average regional homogeneity in the amnestic mild cognitive impairment patients. Each image represents a slice, with 4 mm distance from the other. 0 is the 0 position of space coordinates. The color bar indicates energy intensity.

**Figure 3.**
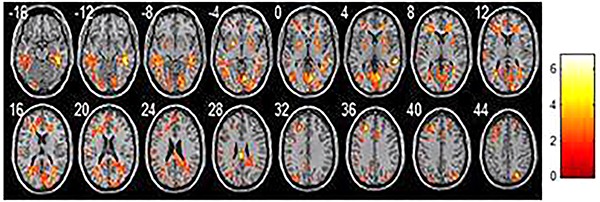
Regions where homogeneity decreased in the amnestic mild cognitive impairment group compared with the normal elderlies. Each image represents a slice, with 4 mm distance from the other. 0 is the 0 position of space coordinates. The color bar indicates energy intensity.

**Figure 4.**
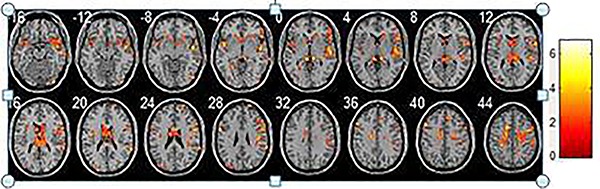
Regions where homogeneity increased in the amnestic mild cognitive impairment groups compared with the normal elderlies. Each image represents a slice, with 4 mm distance from the other. 0 is the 0 position of space coordinates. The color bar indicates energy intensity.

In the resting state, ReHo values were significantly elevated in the posterior cingulate cortex, medial frontal lobe, inner side cortex of the prefrontal lobe, and part of the parietal lobe of normal elderlies and aMCI patients (P<0.05, Figures 1 and 2). Compared with the normal elderlies, ReHo values decreased in aMCI patients in the left temporal lobe (middle temporal gyrus and inferior temporal gyrus), left parahippocampal gyrus, occipital lobe, lingual gyrus, precuneus, and other regions (P<0.05; Figure 3, Table 1), while ReHo values increased in the right side frontal lobe (inferior frontal gyrus), left superior temporal gyrus, precentral gyrus (frontal lobe), right thalamus, the left fusiform gyrus, and other regions (P<0.05; Figure 4, Table 2).

**Table 1.** Regions of the brain with decreased regional homogeneity (ReHo) values in amnestic mild cognitive impairment patients compared with the normal elderlies (P=0.006).

Brain region	Voxel	MNI coordinate			BA
		X	Y	Z	
Fusiform region	8	21	−63	−12	19
Frontal lobe	5	15	27	−6	47
Middle temporal gyrus	1	−57	−63	−3	37
Inferior frontal gyrus	8	54	18	0	45
Parietal lobes	32	0	−69	36	23

**Table 2.** Regions of the brain with increased regional homogeneity in the amnestic mild cognitive impairment (aMCI) group compared with the normal group (P=0.008).

Brain region	Voxel	MNI coordinate			BA
		X	Y	Z	
Parietal lobes	42	0	−69	36	17
Fusiform region	11	21	−63	−12	19
Prefrontal lobe	6	−18	21	−9	47
Inferior frontal gyrus	28	54	18	0	44

## Discussion

MCI is universally recognized as an intermediate state between normal aging and dementia. Since changes in regional cerebral blood flow and metabolism can occur in the early stage of AD, the application of functional imaging technology is helpful for the early diagnosis of AD. Studies have indicated that BOLD-fMRI is a key technique to study the neural mechanism of MCI, since it is able to detect changes in brain functions before MCI develops into AD behavior (3).

In the present study, ReHo analysis of the normal elderlies and aMCI patients revealed that in the resting state, ReHo values were significantly higher in the posterior cingulate gyrus, the inner side of the frontal lobe, the inner side cortex of the prefrontal lobe, and part of the parietal lobe. These brain areas have active functions that reflect the existence of a spontaneous default network in the resting state of the brain. This result is highly consistent with the results from the study of Raichle et al., who found that the exchange of basic information between nerve cells in the brain takes up most of the energy, and a specific task such as memory only accounts for a small part of energy consumption increase. In addition, there are still spontaneous, organized, and continuous brain activities in the resting state. Furthermore, BOLD signals have a high temporal correlation, especially in the inner side cortex of the prefrontal lobe, posterior cortex of the cingulate gyrus, and the outer parietal lobe. Therefore, the concept of a default network was put forward (8).

The present study suggests that the default network is composed of negatively activated brain regions that appear in tasks in a cognitive state, and these brain areas interact with each other to maintain a variety of cognitive activities in the resting state. Research has shown that negative activation is the mechanism of processing the re-allocation of resources (9), assuming that the “resting” state is an organized state of brain activities. When external tasks occur in the brain, these organized processes will be disrupted, related processing resources would be transferred to relevant brain areas associated with the external task, and areas with reduced processing resources would be negatively activated. In addition, Qi et al. (10) demonstrated that abnormality existed in the default mode network of aMCI patients. Moreover, the default-mode network also showed greater abnormalities when compared with high-order visual processing systems in aMCI patients (11).

Compared to the normal elderlies, ReHo values decreased in aMCI patients in the left temporal lobe (middle temporal gyrus and inferior temporal gyrus), left parahippocampal gyrus, occipital lobe, lingual gyrus, precuneus, and other regions. Patients with AD have severe episodic memory impairment due to damage to the storage of information, which is caused by the atrophy of the hippocampus and medial temporal lobe. Episodic memory impairment is the key symptom for the diagnosis of AD. At present, it is considered that the medial temporal lobe (including the entorhinal cortex, the side olfactory cortex, the side hippocampal cortex, and the hippocampus), the prefrontal lobe, and the precuneus are important areas related to episodic memory (12). It was found in the present study that ReHo values in the precuneus and medial temporal lobe were significantly lower than that in the control group. This is similar to previous research results (13,14). Furthermore, Tau protein deposition and magnetic resonance results revealed that changes in atrophy degree is along the pathway of hippocampal atrophy (entorhinal cortex and hippocampus), and is consistent with the decline of early memory (15,16). The medial temporal lobe may be the central area associated with episodic memory. When the patient has not yet developed into AD, time-series homogeneity of BOLD signals in the medial temporal lobe system is decreased (17–19); therefore, the medial temporal lobe can be used as a target to distinguish MCI patients from normal elderlies. In addition, ReHo values significantly decreased in some brain areas of the left inferior temporal gyrus and middle temporal gyrus suggesting that in addition to the medial temporal lobe, the outer portion of the left temporal lobe may also be closely correlated to episodic memory.

Episodic memory disorders are the outstanding manifestations of AD patients; language barriers also exist. Difficulties in finding words and the mild decline in understanding ability occur in the early stage, and the abilities of reading and writing decline with the development of the disease. The results of a study indicated (20) that semantic dementia mainly involves the temporal lobe structure, especially the new cortex in the temporal lobe, which manifest semantic memory disorders, anomia, empty speech, and lack of rational words. This is consistent with the results of the present study, in which ReHo values decreased in the outer portion of the left temporal lobe suggesting that the temporal lobe structure contributes to abnormal brain function in patients with aMCI.

In addition, visual spatial structure dysfunction can occur during the early stage of AD. This visual spatial structure dysfunction is often associated with parietal occipital lobe lesions. We found that in addition to the medial temporal lobe system, ReHo value significantly decreased in the right occipital lobe and lingual gyrus suggesting the presence of resting-state brain functional abnormalities in multiple brain areas, which are associated with memory in aMCI patients. This provides a good starting point for subsequent brain functional connectivity.

It was also found in this study that, compared with the normal group, ReHo value of aMCI patients increased and concentrated mainly in the right frontal lobe, left fusiform gyrus, inferior frontal gyrus, superior temporal gyrus, and right thalamus. These areas are associated with the language center and visual word area, which are closely correlated with the working memory. The episodic memory of AD patients tends to be severely damaged, while abstract thinking and computing power are often not affected during the early stage. Studies have shown that when aMCI patients implemented certain tasks, there were activation performances in the frontal lobe, fusiform gyrus, and other brain areas (21,22). There may be some kind of compensatory mechanism in these areas in patients. Furthermore, aMCI patients in the early stage manifested with episodic memory impairment, while executive functioning was relatively well preserved, which was consistent with the result that the ReHo value increased in these related brain areas in this study. Moreover, this further confirms the compensation hypothesis. This compensatory mechanism would likely slow down the progression of the disease.

Other methods have also been used in the diagnosis of aMCI. Structural MRI can show that gray matter atrophy is particularly severe in patients with MCI that progresses to AD, compared with those whose condition is less severe (23,24). Therefore, gray matter atrophy is effective for predicting whether MCI will deteriorate into AD, but only when the disease has progressed to a certain extent, and patients are not at the early stage. Thus, it is not a good method for early detection of aMCI. Increased white matter abnormalities detected by diffusion tensor imaging have also been used to reflect aMCI (25,26). However, results of different studies are inconsistent, which may be due to the disease stage, as single domain aMCI patients only have memory impairment, but multi-domain aMCI patients have executive function impairment that is related to the frontal lobe (25). Many studies used fMRI on aMCI patients during brain activities, such as associative memory encoding and recognition task, when the brain is not at resting state. The results of such methods are probably different between aMCI subtypes, because whole-brain analysis showed that the active brain regions between multi-domain aMCI and single domain aMCI patients are different (27). Compared with these methods, ReHo evaluation in resting-state fMRI can help us understand the characteristics of the brain neural network and its functions at the resting state in aMCI patients, and thus identify early aMCI to provide treatment at the best time point for the disease.

In summary, an fMRI study was performed on aMCI patients with the application of magnetic resonance equipment. Understanding the characteristics of the brain neural network and its functions at the resting state in aMCI patients can not only help identify aMCI at the early stage but can also provide treatment for this disease at the best time window. In addition, it is also of great significance to have a thorough understanding of brain functions.
